# Relationship between *Helicobacter pylori* infection and gastrointestinal microecology

**DOI:** 10.3389/fcimb.2022.938608

**Published:** 2022-08-18

**Authors:** Wenting Xu, Liming Xu, Chengfu Xu

**Affiliations:** ^1^ Department of Gastroenterology, The First Affiliated Hospital, Zhejiang University School of Medicine, Hangzhou, China; ^2^ Department of Pathology, The First Affiliated Hospital, Zhejiang University School of Medicine, Hangzhou, China

**Keywords:** *Helicobacter pylori*, gastrointestinal microecology, probiotics, infection, eradication

## Abstract

The prevalence of *Helicobacter pylori (H. pylori)* infection has exceeded 50% worldwide, and it is considered a high-risk factor for chronic gastritis, peptic ulcer, gastric adenocarcinoma, gastroesophageal reflux disease and functional dyspepsia. *H. pylori* drug resistance is a common problem worldwide. In recent years, the relationship between *H. pylori* infection and gastrointestinal microecology has received much attention. *H. pylori* infection changes the structure and composition of gastrointestinal microflora by regulating the gastrointestinal microecological environment, local pH value, cytokines and antimicrobial peptides, and immune response and then plays a crucial role in the occurrence and development of digestive system tumors, liver metabolism and extragastrointestinal diseases. The quadruple strategy of *H. pylori* eradication can also aggravate gastrointestinal microflora disorder. However, probiotics can reduce intestinal flora changes and imbalances through different mechanisms, thus enhancing the efficacy of *H. pylori* eradication therapy and reducing adverse reactions caused by eradication therapy. Therefore, this paper reviews the relationship between *H. pylori* infection and gastrointestinal microecology and its clinical application, providing a basis for clinical treatment.

## 1 Introduction

*H. pylori* is a microaerophilic gram-negative bacillus that colonizes the surface of the human gastric epithelium. It has a spiral-curved shape and flagella, which are available for motility (Warren and Marshall, 1983). It is considered a high-risk factor for chronic gastritis, peptic ulcer, gastric adenocarcinoma, gastric mucosa-associated lymphoid tissue lymphoma, gastroesophageal reflux disease and functional dyspepsia (Manabe et al., 2022; Zavros and Merchant, 2022). An epidemiological survey shows that the *H. pylori* infection rate has exceeded 50% worldwide, the infection rate in developed countries is approximately 30%, and it can be as high as 80% in developing countries (Jiang et al., 2021). The average *H. pylori* infection rate in China was 40% in the period 2015-2019 (Ren et al., 2022). The infection rate of *H. pylori* is related to economic situation, living conditions, sanitary conditions and living habits, occupation and quality of drinking water. It has a higher infection rate in situations of poor economic conditions, crowded living, poor sanitary conditions and bad living habits. In different populations, *H. pylori* is observed throughout life in the absence of appropriate treatment, and spontaneous clearance of the infection is very rare (Saito et al., 2021).

The stomach is now thought to contain more than 100 species of bacteria, and *Streptococcus*, *Prevotella*, *Porphyromonas*, *Neisseria* and *Haemophilus* are the dominant bacterial genera in the gastric mucosa, accounting for 70.5% of the total amount (Chen et al., 2021). Slightly different from the bacterial flora of gastric mucosa, the main bacteria in gastric juice of healthy people are ranked from high to low in abundance: *Firmicutes*, *Proteobacteria*, *Bacteroides* and *Actinobacteria*, among which *Streptococcus* and *Rod-like bacteria* of *firmicutes*; *Helicobacter*, *Haemophilus* and *Neisseria of Proteobacteria*; *Prevotella* of *Bacteroidetes*; and *Roche, Actinomycetes* and *Micrococcus* of *Actinomycetes* are the most widely distributed. The composition of the bacteria in the stomach is different from that in the mouth and throat, which suggested that the bacteria in the stomach were not migratory from upper regions. The gastric flora is affected by many factors. Compared with the intestinal flora, the gastric flora is characterized by low density, low specificity, poor stability and high fluctuation. According to previous research results, great differences were observed in the weight and number of bacteria in the gastric microecosystem of healthy adults, which might be due to the different influencing factors, such as regional span, cultural difference, dietary habit difference, etc. (Stewart et al., 2020). These differences may also be related to the age and sex of the population being studied, the method of testing the samples, the use of antibiotics, and whether other diseases were present.

The number of gut microbes is extremely large and includes bacteria, archaea, fungi, viruses, etc. These microorganisms colonize the gut after a series of complex processes, form relatively stable communities, and interact with the host to form a unity with a mutually beneficial symbiotic relationship. The gut microbial genome contains approximately 3.3×10^6^ nonrepetitive genes, which is 150 times the number of genes in the human genome and considered the second genome of the human body. Harmful bacteria, opportunistic pathogens and probiotics are the three main flora in the gut. More than 99% of intestinal microorganisms are anaerobic bacteria, mainly including *Firmicutes, Bacteroidetes, Proteobacteria, Actinobacteria, Fusobacterium, Verrucobacterium, Cyanobacteria* and *Spirochetes*, the most important of which are *Firmicutes* (50% to 70%) and *Bacteroidetes* (10% to 30%) (Lensu and Pekkala, 2021). Its function is closely related to human health and not only affects the nutrient supply and energy balance but also participates in immune defense, endocrine regulation, barrier function maintenance, inhibition of pathogen colonization and many other physiological processes (Ruch and Engel, 2017). To identify the core composition pattern of human gut microbiota in a healthy state, Arumugam *et al.* proposed the concept of “enterotype” and believed that the composition of human gut microbiota could be divided into three types: “Enterotype 1” was mostly *Bacteroides*, “Enterotype 2” was mostly *Prevotella*, and “Enterotype 3” is mostly *Ruminococcus* (Arumugam et al., 2011). The three types represent three different symbiotic relationships between gut flora and the host and commonly do not change based on differences in race, sex, age, body weight, and health status. Once any kind of bacteria in the intestinal tract exceeds the normal range and reaches a certain pathogenic dose or changes in species and locations, *etc.*, it will lead to dysbiosis. In recent years, studies have found that dysbiosis of intestinal flora may cause systemic lupus erythematosus, rheumatoid arthritis, diabetes, obesity, autism, multiple sclerosis and other extraintestinal system diseases as well as intestinal system diseases, such as inflammatory bowel disease and colon cancer (Jackson and Theiss, 2020; Scheithauer et al., 2020; Tomofuji et al., 2022).

**Figure 1:**
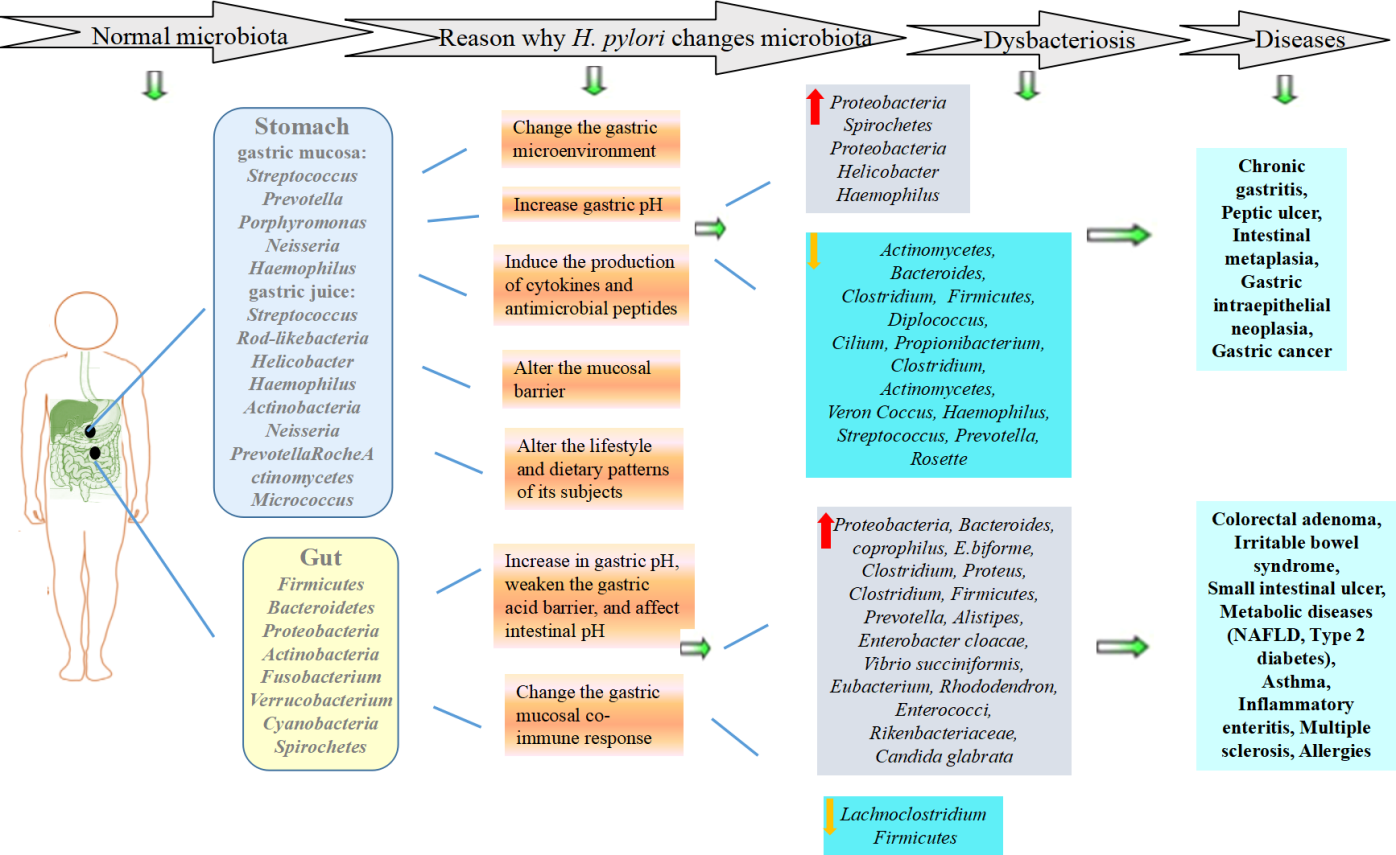
Effects of *H. pylori* infection on gastrointestinal microecology and diseases.

*H. pylor*i infection can affect the gastrointestinal microecological environment, leading to disruption of biological barriers and bacterial translocation, which plays a common role in the occurrence and development of diseases (Lapidot et al., 2021) (**Figure 1**). In addition, *H. pylori* drug resistance is a common problem worldwide (Kuo et al., 2021). Probiotics can be divided into single strains and mixed strains, and probiotics represents the general term for beneficial bacteria living in the intestinal tract. They play an important role in the eradication of *H. pylor*i, which also provides new ideas for the treatment of *H. pylor*i infection. Therefore, this paper reviews the relationship between *H. pylor*i infection and gastrointestinal microecology and the effect of their interaction on diseases to provide a basis for clinical treatment.

## 2 Effects of *H. pylori* infection on gastrointestinal microecology and diseases

### 2.1 *H. pylori* infection and gastric microecology

*H. pylori* dominates gastric mucosa-associated flora, and excessive proliferation of *H. pylori* may interfere with the gastric microecological environment, resulting in microecological disorders and inflammatory reactions, resulting in disordered gastric microflora and the occurrence of diseases. Studies have shown that *H. pylori* significantly reduces the diversity of the microbiota in the stomach. The changes in the proportion and genus level of each phylum are mainly manifested in increases in the relative abundance of *Proteobacteria* and *Spirochetes* but decreases in the abundance of *Actinomycetes*, *Bacteroides*, *Clostridium* and *Firmicutes* in the gastric mucosa. The relative abundance of *Proteobacteria* in the gastric juice increases, while the relative abundance of *Diplococcus*, *Cilium*, *Propionibacterium*, *Clostridium*, *Actinomycetes*, *Veron Coccus*, *Haemophilus*, *Streptococcus* and *Prevotella* decrease. (Bakhti and Latifi-Navid, 2021). At the genus level, *Helicobacter* and *Haemophilus* increase while *Rosette* decreases (Duan et al., 2022). This phenomenon further shows that *H. pylori* has advantages over other flora. It is not clear why *H. pylori* infection changes the gastric microecology, although possible reasons have been postulated (Cheok et al., 2021): *H. pylori* changes the gastric microenvironment, and the ammonia and bicarbonate produced by decomposing urea can also serve as substrates for other microbial communities; *H. pylori* infection induces the regulation of the H+/K+-ATPase D subunit promoter by the Nuclear Factor- Kappa B (NF-kB) P50 homologous dimer, which downregulates the expression of H+/K+-ATPase mRNA, and inhibits gastric acid secretion, thereby increasing gastric pH and creating favorable conditions for the colonization of other microorganisms; *H. pylori* can induce the production of cytokines and antimicrobial peptides, thereby leading to chronic gastritis and inhibiting other local microorganisms; *H. pylori* may directly alter the mucosal barrier by changing the expression of gastric mucin; *H. pylori* can alter the lifestyle and dietary patterns of its subjects; *H. pylori* can develop T regulatory-mediated tolerance to other bacterium, and suppress the effector T-cell responses (Sung et al., 2020; Marginean et al., 2021).

**Figure 2:**
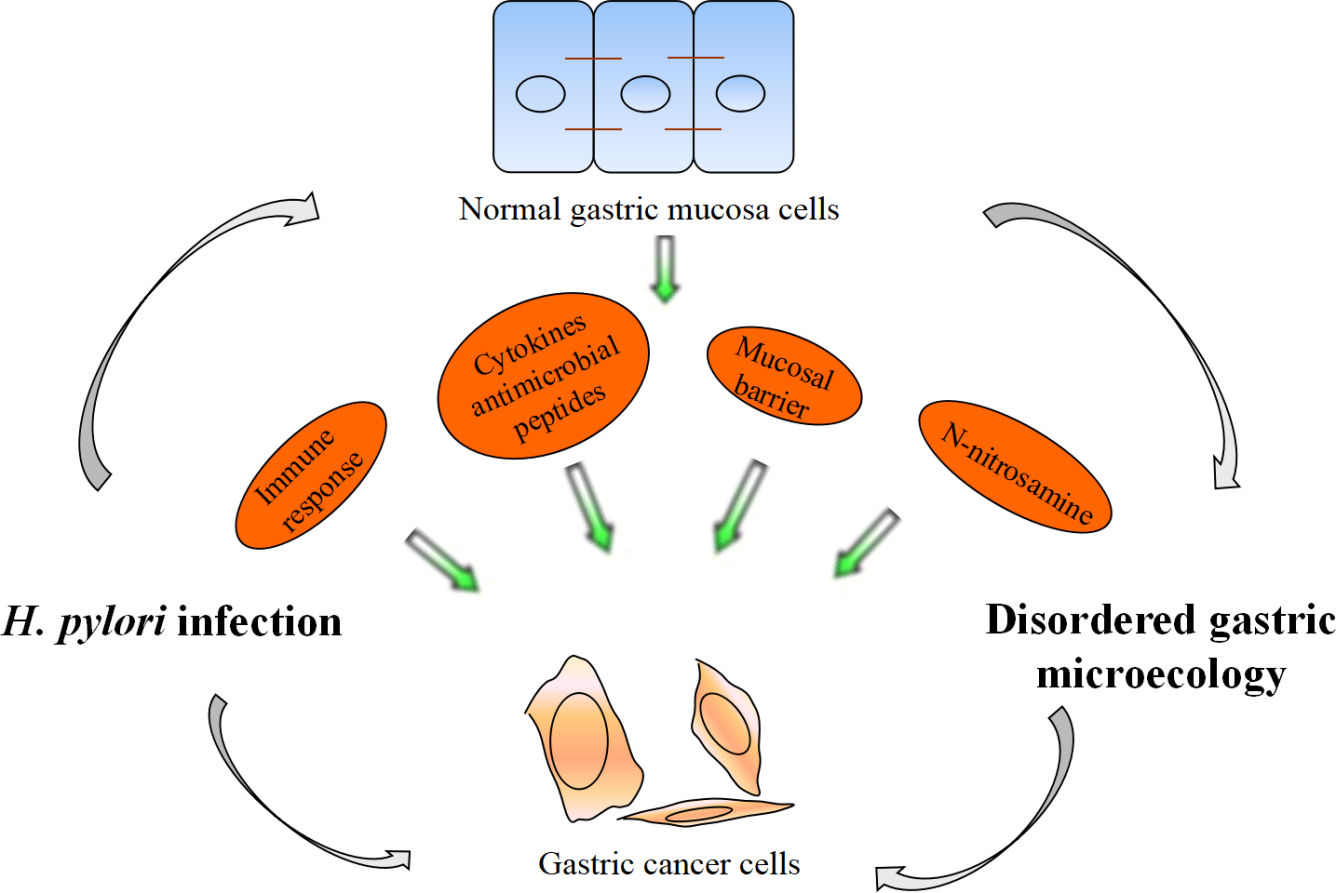
*H. pylori* infection promotes the occurrence of gastric cancer by regulating gastric flora.

Recent research and practice have shown that *H. pylor*i infection is the main cause of the occurrence and development of chronic gastritis, peptic ulcer, gastric cancer and other digestive diseases. The specific mechanism is still not completely clear, but changes in gastric microecology may be a main reason. *H. pylor*i infection affects Th1-related IgG2 immune response, Interleukin (IL)-1β, IL-17 and Reg III γ transcription levels in the stomach and reactive oxygen species (ROS) levels by gastric flora, promoting the occurrence of superficial gastritis, atrophic gastritis, intestinal metaplasia and gastric intraepithelial neoplasia (Doulberis et al., 2021; Araujo et al., 2022). *H. pylor*i infection promotes the occurrence of gastric cancer by regulating gastric flora (**Figure 2**). The next generation gene profile analysis of gastric microbiome composition showed that compared with the gastric microbiome of patients with chronic gastritis, gastric cancer patients had a microbiome imbalance characterized by lower diversity, lower *H. pylori* abundance and higher genotoxicity (Ferreira et al., 2018). Chronic *H. pylor*i infection results in decreased gastric acid secretion, and non-*H. pylori* microbiota overgrowth promotes gastric mucosa malignancy by promoting inflammation, stimulating cell proliferation, changing stem cell dynamics and transforming nitrate into n-nitrosamine. In gastric cancer, the microbiota promotes nitrite reduction, and acidic nitrite can kill other bacteria. Nitrate can be used as an energy source and can change the gastric microbiota, causing ecological disorder. N-nitroso compounds formed during nitrite metabolism are important carcinogens. In addition, they release DNA-damaging reactive oxygen species and nitrogen (they are effective mutagens), continuously aggravating the gastric mucosal inflammatory response, accelerating the progression of gastric precancerous lesions, and promoting the occurrence of gastric adenocarcinoma (Sheweita and Alsamghan, 2020). *H. pylor*i infection increased *Escherichia coli*, *Lactobacillus*, nitrate reductase bacteria, *L. coleohominis* and *Spirobacter nitroso* in gastric cancer and played a role in nitrite metabolism. Other bacteria, such as *Clostridium*, *Hemophilus*, *Staphylococcus* and *Neisseria*, may also be involved in the formation of these compounds (Engstrand and Graham, 2020; Liu et al., 2021). In addition, genomic DNA of *Pseudomonas* can be transferred into human somatic cells through some mechanism, thus upregulating proto-oncogene expression and promoting the occurrence of gastric adenocarcinoma (Rodrigues et al., 2019). *Lactobacillus* can promote immune tolerance and provide a platform for the colonization of other carcinogenic bacteria (Chen et al., 2021).

### 2.2 *H. pylori* infection and intestinal microecology

Studies have shown that *H. pylori* infection can not only affect the gastric microecology but also change the intestinal flora. The relationship between *H. pylori* infection and intestinal microecology is still not clear, and the conclusions in the literature are controversial (Fujimori, 2021). A study on children showed that the abundance and diversity of gastrointestinal flora were higher in children without *H. pylori* infection, among which *Proteobacteria, Bacteroides, Clostridium, etc., w*ere more abundant (Llorca et al., 2017). However, another study found that compared with uninfected children, *H. pylori*-positive children had an increased number of intestinal flora, such as *Proteus, Clostridium, Firmicutes*, and *Prevotella* (Kakiuchi et al., 2021). Lu found that the diversity of intestinal flora in *H. pylori*-infected patients was higher, in which the abundance of *Alistipes and Enterobacter cloacae* increased and the abundance of *Lachnoclostridium* decreased. *H. pylori* infection changes the balance of intestinal microecology (He et al., 2019). Researchers analyzed human feces and found that the diversity and complexity of the intestinal flora of *H. pylori*-infected patients were higher, in which *Vibrio succiniformis*, *Rhododendron* spp.*, Enterococci* spp., and *Rikenbacteriaceae* spp. increased and the abundance of *Candida glabrata* and unclassified fungi improved (Dash et al., 2019). The underlying reason may be that *H. pylori* infection leads to the initiation of the mucosal coimmunity response as well as changes in gastric acid and gastrin secretion, resulting in an increase in gastric pH, weakening the gastric acid barrier, and simultaneously affecting intestinal pH, which in turn promotes changes in intestinal flora (Iino and Shimoyama, 2021). *H. pylori* infection may also change the distal intestinal flora through mucosal co-immune response by cytotoxin-associated gene A (CagA), Vacuolating cytotoxin A (VacA), which impairs cell polarity and affects host signaling pathways, thereby altering immune phenotypes and promoting inflammation (He et al., 2022).

An imbalance in *H. pylori*-related intestinal flora can lead to the occurrence of nonalcoholic fatty liver disease (NAFLD), colorectal adenoma, irritable bowel syndrome and small intestinal ulcer, and the mechanism may be that *H. pylori* invades the intestinal mucosa to increase intestinal permeability and leads to an imbalance of intestinal flora, which facilitates the entry of bacterial endotoxins into the liver through the portal vein, thereby promoting intestinal inflammation (Santos et al., 2020; Fujimori, 2021). It also affects the absorption of iron and vitamin B12 in the intestinal tract and the metabolic process of carbohydrates and amino acids in the host (Santos et al., 2020). *H. pylori* infection can increase *Eubacterium*, *Bacteroides coprophilus*, *E.biforme* and decrease *Firmicutes*, which are related to high density lipoprotein (HDL)/low density lipoprotein (LDL) ratio (Martin-Nunez et al., 2020). Meanwhile, *H. pylori* infection can affect the balance of gastrointestinal hormones such as leptin, ghrelin and gastrin, and alter the fermentation of non-digestible carbohydrates, the generation of short-chain fatty acids (SCFAs) and glucagon-like peptide-1 (GLP-1) through intestinal flora and may be involved in the occurrence of metabolic diseases such as insulin resistance, and type 2 diabetes (Martin-Nunez et al., 2021; Hu et al., 2022). In addition, *H. pylori* infection affects the intestinal microecosystem and can also lead to diseases of the extraintestinal system. *H. pylori*-induced changes in intestinal microflora were often accompanied by changes in immune-related gene expression in multiple organs. Changes in gut microecology and microenvironment caused by *H. pylori* infection (e.g., bacterial diversity and changes in gastric pH) affect the expression of some immune-related genes in the host, and changes in lung-related gene expression may be caused by *H. pylori* infection which promoting cumulative changes in immune and/or inflammatory responses. These changes can also be observed at distant sites in host organisms, and other members of the genus *Helicobacter* exhibit similar effects. For example, the natural colonization of liver *Helicobacter pylori* in the digestive tract of mice can lead to the transformation of intestinal microbiota, resulting in subclinical inflammation and severe immune damage (Li et al., 2020). Other studies found that intestinal flora disorders caused by *H. pylori* colonization can also play a protective role in some diseases through immune regulation, such as asthma, inflammatory enteritis, and multiple sclerosis (Borbet et al., 2019). Early *H. pylori* infection-induced changes in the microenvironment affect the expression of some immune-related genes in the host and then affect the local and even systemic immune system. The effect of *H. pylori* on the immune system is closely related to changes in gastric physiology and microflora and the occurrence and development of extragastric diseases. *H. pylori* affects the adaptive immune response by interfering with antigen presentation and T-cell response regulation through microecological imbalance, which plays an important role in the prevention of extragastric immune and inflammatory diseases (such as childhood asthma and allergies) and metabolic disorders (Zuo et al., 2021). *H. pylori* can regulate the levels of Treg cells and cytokines such as IL-10 and transforming growth factor β (TGF-β) in the lung through intestinal flora, prevent or regulate the Th2 response to allergens, and induce the allergen-specific immune response to immune tolerance transition. This prevents the development of asthma and other allergic diseases (Borbet et al., 2019). In addition, *Tricorniaceae* can reduce the colonization of *Clostridium difficile* and reduce the occurrence of intestinal inflammation (Castro-Fernandez et al., 2019). From the influence of *H. pylori* on disease, we can see that *H. pylori* has a complex effect on the host.

### 2.3 *H. pylori* eradication gastrointestinal microecology

Presently, a consensus report on the management of *H. pylori* infection recommends the use of quadruple therapy consisting of proton pump inhibitor (PPI), bismuth and two antibacterial drugs for 10 or 14 days (Liou et al., 2020). However, *H. pylori* eradication quadruple therapy has a certain impact on gastrointestinal flora (Guo et al., 2020). The powerful acid-suppressing effect of PPI can not only reduce the removal effect of exogenous bacteria in food by gastric acid and weaken the body’s own natural barrier but also weaken the body’s digestive function (Li et al., 2017). However, macromolecular nutrients that are not fully digested may promote the growth of pathogenic bacteria after entering the intestinal tract, which has a significant impact on the composition of the flora. In addition, PPI can also reduce the viscosity of gastric mucus, prolong gastric emptying time, and then induce the destruction of gastric microecological balance, resulting in a significant decrease in the abundance and diversity of commensal bacteria and increasing the growth of harmful bacteria such as *Motococcaceae*, *Oxidobacteriaceae* and *Sphingobacteriaceae*, *Streptococcaceae*, *Veronococcaceae*, *Ruminococcaceae*, *Microococcaceae*, *Flavobacteriaceae* and *Enterococcaceae*, thus resulting in osteoporosis, electrolyte imbalance, small intestinal bacterial overgrowth and *Clostridium difficile* infection (Le Bastard et al., 2021). Individuals with small intestinal bacterial overgrowth have no obvious symptoms in a short period of time, but bacterial overgrowth can lead to excessive fermentation of carbohydrates in the intestinal lumen and reduced absorption of iron, vitamin B12 and fat, which can lead to sequelae, such as abdominal distension in the later stage (Weitsman et al., 2022).

In addition, the long-term use of antibiotics is another important cause of gastrointestinal flora imbalance, damaging the gastrointestinal flora biological barrier, chemical barrier and immune barrier. The breakdown of the biological barrier manifests as a reduction in the number of antibiotic-sensitive bacteria and an increase in the number of drug-resistant bacteria. Then, the latter becomes the dominant flora. Therefore, it destroys the colonization ability of normal symbiotic flora, and exogenous pathogenic bacteria are more likely to invade the body (Zhou et al., 2021). *Bacteroidetes, Bifidobacterium*, *Clostridium*, *Enterobacteriaceae and Lactobacillus* are the most susceptible flora, and opportunistic pathogens such as *Escherichia coli, Proteus, Morganella*, *Serratia*, *Streptococcus, Shigella, Klebsiella pneumoniae, etc.*, increase and replace the above normal flora to become the dominant flora. SCFA producing bacteria such as *Lachnospiraceae, Ruminococcaceae, Eubacteriacea, Bacteroides, Faecalibacterium, Roseburia, Phascolarctobacterium* also decrease with the short-term of the antibiotic treatment (Guillemard et al., 2021). The destruction of the chemical barrier can cause the intestinal microbiota to produce the enzyme cytochromatin (P450), which affects the oxidative metabolism of food and oral drugs (Jonaitis et al., 2020). Disruption of the immune barrier can affect gastrointestinal antigen presentation and innate immunity. The degree of influence of antibiotics on gastrointestinal microecology is related to various factors, such as types, doses, courses of treatment, routes of administration, and microbial resistance. The extensive and unreasonable application of antibiotics is the main reason for the increase in *H. pylori* drug resistance and the failure of eradication therapy (Megraud et al., 2021). Antimicrobial-associated gastrointestinal flora imbalance can cause a variety of clinical manifestations. The most common adverse reactions include diarrhea, nausea, vomiting, abdominal distension, and abdominal pain, which can increase the risk of the interruption and failure of treatment and occurrence of drug-resistant strains. Antimicrobial-associated gastrointestinal dysbiosis may also affect the long-term prognosis of the disease. The recovery time of gastrointestinal flora after antibiotics was also related to the type of antibiotics, and multiflora could recover to the level before treatment within 4 weeks; however, in some patients, the microbiota was in an “irritable” state for a long time after treatment, and it could take up to 4 years to fully recover to pretreatment levels (Jakobsson et al., 2010; Suzuki et al., 2021). At present, there is still a lack of sufficient clinical evidence for the effects of different *H. pylori* eradication regimens on gastrointestinal microecology. A good experimental design and standardized operation are the keys to obtain reliable research data.

## 3 Effect of intestinal probiotics on *H. pylori* eradication

**Figure 3:**
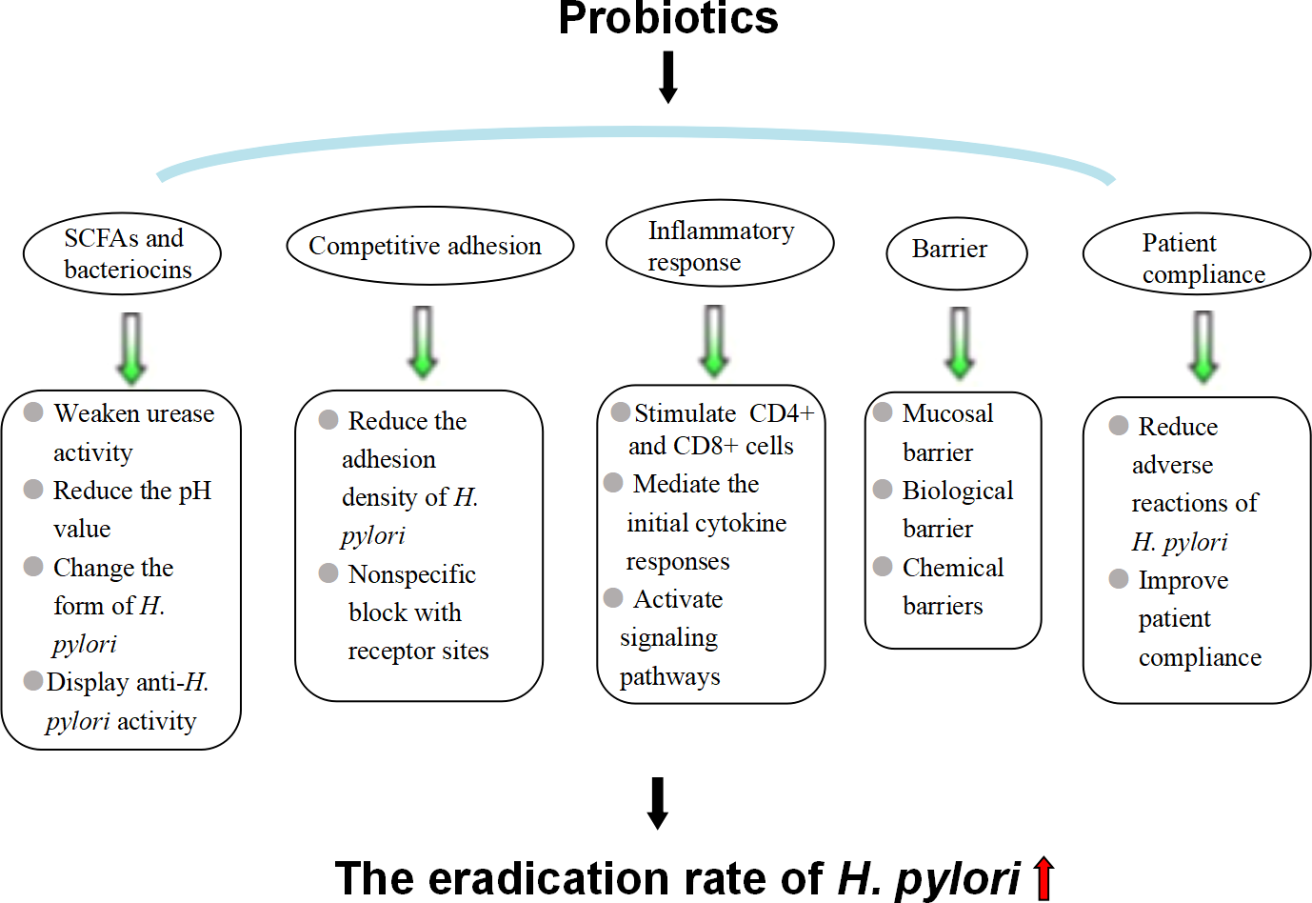
Mechanism by which probiotics improve the eradication rate of *H. pylori.*.

Intestinal probiotics are the general term for the original beneficial bacteria in the gut. Common probiotics include (1) *Lactobacillus: L. helveticus, L. acidophilus, L. brevis, L. casei, L. johnsonii, L. rhamnosus, etc.;* (2) *Bifidobacterium; (3) Streptococcus: Streptococcus thermophilus, Streptococcus faecalis, Lactococcus, Streptococcus intermedia, etc.;* and (4) *yeast bacteria*. Studies have shown that probiotic preparations combined with antibiotics can effectively relieve the clinical symptoms of patients with *H. pylori* infection, improve the effect of *H. pylori* eradication and reduce the incidence of adverse drug reactions (Baral et al., 2021) (**Figure 3**). Commonly used probiotic preparations in the clinic include *L. acidophilus, Bacillus licheniformis, Bifidobacterium triple viable bacteria*, Bacillus subtilis dual viable bacteria and Saccharomyces boulardii (Lu et al., 2022).

### 3.1 Probiotics improve the *H. pylori* radical cure effect

The compound *L. acidophilus* tablet is a compound tablet composed of four kinds of bacterial powder, including the Chinese strain *L. acidophilus*, Japanese strain *L. acidophilus, Streptococcus faecalis* and *B. subtilis*, and it represents a kind of intestinal flora adjustment drug (Ji et al., 2018). The main mechanism of its influence on *H. pylori* is mainly as follows: it secretes bacteriocin, lactic acid, hydrogen peroxide and other substances with antibacterial effects, inhibits the proliferation and growth process of *H. pylori*, and plays a role in eliminating *H. pylori*; it secretes some active substances against bacterial adhesion, leading to a decrease in the adhesion index of *H. pylori* to gastric epithelial adenocarcinoma cells; and it upregulates the expression level of small intestinal mucosal binding proteins, indirectly promoting the recovery of gastric mucosal permeability; and it inhibits the release of inflammatory factors, thereby inhibiting its pathogenicity (Takagi et al., 2016; Ji et al., 2018). *Compound L. acidophilus* combined with quadruple therapy improves the eradication rate and reduces the incidence of adverse reactions compared with quadruple therapy alone and can serve as a rescue regimen in cases of treatment failure (Karbalaei and Keikha, 2021).

*B. licheniformis* can treat bacteria by prompting the body to produce antibacterial active substances and kill pathogenic bacteria. After entering the digestive tract, *B. licheniformis* has an antagonistic effect on the pathogenic bacteria in the gastrointestinal tract while promoting the growth of beneficial flora to regulate the flora of the digestive tract. In addition, *B. licheniformis* can also cause a hypoxic environment in the digestive tract, which is conducive to the growth of anaerobic bacteria (Asaka et al., 2014). These two effects can not only regulate the function of the gastrointestinal tract but also improve adverse reactions during the treatment process, thereby preventing and treating gastrointestinal diseases (Feng et al., 2022). Therefore, the use of *B. licheniformis* to intervene in *H. pylori* eradication has played a positive role in the treatment.

*Bifidobacterium triple viable bacteria* is a live bacterial preparation made of three probiotics in an appropriate proportion. It is mainly composed of *Bifidobacterium*. The three together form a combined flora that can grow under different conditions. After entering the gastrointestinal tract, *Bifidobacterium triple viable bacteria* can reduce the pH value of the lesions, inhibit the growth of harmful bacteria, promote the reproduction of beneficial bacteria, and adjust the balance of the body flora (Di Pierro et al., 2020). *Bifidobacterium triple viable bacterial* capsules reduce the incidence of adverse reactions in the treatment of *H. pylori*-related gastritis and peptic gastric ulcer, and the clinical efficacy and eradication rates of gastritis and ulcers are significantly better than those of traditional therapies (Koretz, 2018).

*B. subtilis dual live bacteria* are composed of two probiotics, mainly *B. subtilis*, which can stimulate the release of active bacteria, such as *Candidatus Soleaferrea*, increase the activity of the body’s own beneficial bacteria, improve the immune function of the intestinal tract, and inhibit the reproduction of pathogens in the intestinal tract, such as *Lachnospiraceae, Ruminiclostridium, Lachnospiraceae* and *Oxalobacter*, thereby regulating intestinal flora (Zou et al., 2022). *B. subtilis dual viable bacteria* combined with quadruple therapy can improve the eradication rate of *H. pylori* and reduce adverse reactions.

*Saccharomyces boulardii* is a nonpathogenic yeast with natural resistance to antibacterial drugs, gastric acid and pepsin. It can have antibacterial and anti-inflammatory activities by inhibiting NF-κB from entering the nucleus and can stimulate the mucosal epithelium to secrete secretory IgA (sIgA), thus enhancing the body’s innate immune defense (Zhang et al., 2020).

### 3.2 Mechanism by which probiotics improve the eradication rate of *H. pylori*

#### 3.2.1 SCFAs and bacteriocins

Studies have shown that probiotics and their metabolites can inhibit or kill *H. pylori* (Ge and Zheng, 2012). The substances that probiotics inhibit the growth of *H. pylori* mainly include SCFAs and bacteriocins (Xu et al., 2021). SCFAs, such as formic acid, acetic acid, propionic acid, butyric acid, and lactic acid, are produced by probiotics metabolizing carbohydrates. They inhibit the colonization and growth of *H. pylori* in the gastric mucosa by weakening urease activity, reducing the pH value in the stomach, and changing the form of *H. pylori* (Keikha and Karbalaei, 2021). Bacteriocin is a bactericidal or bacteriostatic substance produced by bacteria that exhibits a narrow activity inhibition spectrum for species of closely related strains. Studies have found that *L. formus, B. subtilis, L. roche, Enterococcus faecalis, B. subtilis and Bifidobacterium* can all release bacteriocin with anti-*H. pylori* activity, such as nisin from Streptococcus lactis, Amicoumacin A, Reuterin, etc., and their anti-*H. pylori* effects depend on the type and number of bacterial strains, which may be related to the diversity of clinical trials (Gotteland et al., 2006). The specific mechanism of bacteriocin: the bacteriocin nisin can kill *H. pylori* under the coordination of citric acid, similar to most bacteriocins. Nisin molecules are positively charged, containing three lysines at locus 12, 22 and 34 and one histidine at locus 31, which is advantageous for electrostatic and hydrophobic interactions with the cell membrane, inserting into the membrane formation, and can lead to cell autolysis and death (Liang et al., 2022). In addition, probiotics can produce substances with a broad spectrum of antibacterial effects, such as hydrogen peroxide, which can resist *H. priori* infection.

#### 3.2.2 Competitive adhesion

Researchers studied the effect of *Lactobacillus* on *H. pylori* adhesion to gastric adenocarcinoma cells, detected the number of *H. pylori* adhering to gastric adenocarcinoma cells –AGS and MKN45 cells by urease test, and found that both live and dead *Lactobacillus* could adhere to AGS cells and MKN45 cells in large quantities, significantly reducing the adhesion density of *H. pylori*, although the inhibitory ability of dead bacteria was lower than that of live bacteria (Rokka et al., 2008; Takeda et al., 2017). *Lactobacillus* can bind to a variety of pathogenic bacterial receptors and inhibit the adhesion of pathogenic bacteria to gastric mucosa epithelial cells (Song et al., 2019; Lee et al., 2020). It is speculated that the nonspecific blocking of receptor sites by *Lactobacillus* may be the mechanism of its inhibition of *H. pylori*. Gastric epithelial cells have *H. pylori*-specific adhesion receptors, such as glycolipid receptor gangliotetracylsphingol and sulfatide. These studies showed that *L. reuteri* and *H. pylori* have common glycolipid specificity, which can secret adhesion factors and competitively inhibit the adhesion of *H. pylori* by binding to the binding site of gastric mucosal epithelial cells.

#### 3.2.3 Inhibiting the inflammatory response to *H. pylori* infection

Probiotics can produce a cellular immune response in gastrointestinal mucosa, especially stimulating the activation and proliferation of CD4+ and CD8+ cells in the mucosa propria, increasing the production of secretory immunoglobulin A, reducing intestinal permeability, promoting the proliferation of epithelial cells, accelerating the regeneration of mucosal repair, and strengthening the role of the mucosal barrier. *H. pylori* stimulates gastric epithelial cells to secrete IL-8, mediating the initial cytokine responses and resulting in neutrophil and monocyte migration to the mucous membrane. Some probiotics have been shown to inhibit CagA protein expression and reduce the *H. pylori*-induced secretion of IL-8 by gastric mucosa epithelial cells, thus inhibiting the inflammatory responses (He et al., 2022). In addition to IL-8, *H. pylor* ***i*** infection can also stimulate epithelial cells to produce a number of other inflammatory mediators, such as tumor necrosis factor α, inducible nitric oxide synthase, and cyclooxygenase-2, while probiotics block nuclear transport of the NF-κB signaling pathway by increasing the expression of cytokine signal transduction inhibitors, activating transcriptional activators and inactivating janus kinase (JAK) to upregulate the expression of suppressor of cytokine signal transduction 2 (SOCS2) or SOCS3 and activate signal transducerand activator of transcription 1 (STAT-1) and STAT-3 inactivation protein tyrosine kinase 2 (JAK2), thereby inhibiting the inflammatory response caused by *H. pylori* infection and inhibiting inflammation (Lee et al., 2010; Song et al., 2019). More research is needed because controversy remains about the underlying mechanism as it may be independent of STAT signaling (Sarajlic et al., 2020).

#### 3.2.4 Strengthening mucosal, biological and chemical barriers to prevent *H. pylori* colonization

Probiotics can indirectly promote the recovery of gastric mucosal permeability, maintain the integrity of the mucosa, prevent the invasion of pathogenic bacteria, including *H. pylori*, and strengthen the mucosal barrier. Probiotics interact closely with mucosal epithelial cells to occupy the mucosal surface and improve the defense ability of epithelial cells, thus forming a biological barrier (Chakravarty and Gaur, 2019). Active substances, such as metabolites of probiotics, including small molecule acids, hydrogen peroxide and bacteritin, can kill pathogenic bacteria, including *H. pylori*, and prevent the colonization and invasion of pathogenic bacteria and opportunistic pathogens, forming a chemical barrier.

#### 3.2.5 Reducing adverse reactions and improving patient compliance

Probiotic preparations can alleviate adverse reactions, such as taste disturbance, diarrhea, nausea, abdominal pain and constipation caused by antibiotics and significantly improving the compliance of patients with *H. pylori* eradication therapy, thereby increasing the *H. pylori* eradication rate (Zagari et al., 2018). Treatment compliance is an important factor in the success of *H. pylor*i eradication (Nyssen et al., 2021). However, previous studies showed that probiotic preparations had the risk of systemic infection, as long-term use of *B. subtilis* carried the risk of cholangitis. Additionally, excessive immune stimulation in susceptible individuals may be induced, as the use of *Saccharomyces cerevisiae* and *Saccharomyces boulardii* improved the risk of fungalemia in patients with prolonged antibiotics or venous catheterization deleterious metabolic activities (Doron and Snydman, 2015). Therefore, careful consideration should be given to the clinical application of probiotics to patients with severe underlying diseases, low immunity, long-term antibiotic use, and central venous catheterization.

## 4 Discussion

In conclusion, *H. pylori* infection can change the structure and composition of gastrointestinal flora by regulating the local microenvironment, local pH value, cytokines and antimicrobial peptides, and immune response and then play a role in the occurrence and development of digestive system tumors, liver metabolism and extragastrointestinal diseases. The quadruple regimen of *H. pylori* eradication can aggravate gastrointestinal microflora disorder. Probiotics can reduce the change and imbalance of intestinal flora and improve the eradication rate of *H. pylori*. However there are still many challenges in this field. (1) The influence of *H. pylori* infection on gastrointestinal microecology is still controversial, and opposite conclusions have been obtained, which may be related to the infection time, host health status, age of research subjects, number of research samples, methods of sampling and detection, analysis from different phyla levels of gastrointestinal flora and other factors. (2) *H. pylori* eradication can restore the gastric acid barrier to a certain extent and then restore the balance of intestinal flora. At the same time, oral administration of large doses of antibiotics and PPIs can easily disrupt the normal symbiosis between the intestinal flora and the host, thereby weakening the protective effect of the biological barrier. Secondary changes in the intestinal flora after eradication may be related to the adverse reactions associated with eradication therapy and the recurrence of infection after eradication. However, it has not been determined whether *H. pylori* eradication causes long-term changes in intestinal flora. (3) The mechanism of *H. pylori* and gastrointestinal microecology in specific diseases and the causal relationship between *H. pylori* and diseases remain unclear. Different subtypes of gastric cancer are identified according to different types of ecological disorders, and personalized probiotic adjuvant therapy is developed. Future research should focus on analyzing bacterial imbalance in the oral cavity and stool as a noninvasive diagnostic marker and preventing and treating gastritis cancer by intervening with bacterial flora and studying its specific mechanism in nutrition, immunity, metabolism, signaling pathway and other aspects and the mechanism of interaction between H. pylori and other gastrointestinal microorganisms in gastric diseases. (4) Many studies have shown that probiotic adjuvant therapy can reduce changes and imbalances in the intestinal flora caused by eradication therapy, including antibiotics and PPIs, thus enhancing the efficacy of eradication therapy and reducing its adverse reactions. During *H. pylori* eradication therapy, probiotics as adjuvants can be used as follows: Try not to choose probiotics containing *Enterococcus* are more likely to develop resistance and resistance genes can be easily passed to *H. pylori* and other pathogenic bacteria by plasmids, which affects *H. pylori* eradication therapy; 2-4 weeks may be the optimal time to use probiotics; the time interval of probiotic and standard antibiotic treatments must be selected carefully so that probiotics can better colonize and play a role. However, homogeneity is difficult to control in research on the relationship between probiotics and effect of *H. pylori* eradication treatment because many factors need to be considered, such as the types of probiotics, dosage forms, types of eradication drugs, courses of treatment, and regional differences. It is necessary to carry out large-scale multicenter clinical studies to effectively improve the eradication rate of *H. pylori* and reduce the incidence of adverse reactions.

## Author contributions

WX and LX wrote the manuscript; CX revised the review. All authors contributed to the article and approved the submitted version.

## Funding

This research was supported by The National Natural Science Foundation of China (No. 82100600), Zhejiang Provincial Natural Science Foundation of China under Grant (No. LQ20H030009), and Health Science and Technology Plan Project of Zhejiang Province (No. 2018KY367).

## Conflict of interest

The authors declare that the research was conducted in the absence of any commercial or financial relationships that could be construed as a potential conflict of interest.

## Publisher’s note

All claims expressed in this article are solely those of the authors and do not necessarily represent those of their affiliated organizations, or those of the publisher, the editors and the reviewers. Any product that may be evaluated in this article, or claim that may be made by its manufacturer, is not guaranteed or endorsed by the publisher.
